# Meta-analysis of genome-wide linkage studies of asthma and related traits

**DOI:** 10.1186/1465-9921-9-38

**Published:** 2008-04-28

**Authors:** Samuel Denham, Gerard H Koppelman, John Blakey, Matthias Wjst, Manuel A Ferreira, Ian P Hall, Ian Sayers

**Affiliations:** 1 Division of Therapeutics & Molecular Medicine, University Hospital of Nottingham, Nottingham, UK; 2 Pediatric Pulmonology and Pediatric Allergology, Beatrix Children's Hospital, University Medical Center Groningen, University of Groningen, The Netherlands; 3 Institute of Epidemiology, Ingolstädter Landstraße 1, D-85764 Neuherberg, Germany; 4 Center for Human Genetic Research, Massachusetts General Hospital, Boston, USA

## Abstract

**Background:**

Asthma and allergy are complex multifactorial disorders, with both genetic and environmental components determining disease expression. The use of molecular genetics holds great promise for the identification of novel drug targets for the treatment of asthma and allergy. Genome-wide linkage studies have identified a number of potential disease susceptibility loci but replication remains inconsistent. The aim of the current study was to complete a meta-analysis of data from genome-wide linkage studies of asthma and related phenotypes and provide inferences about the consistency of results and to identify novel regions for future gene discovery.

**Methods:**

The rank based genome-scan meta-analysis (GSMA) method was used to combine linkage data for asthma and related traits; bronchial hyper-responsiveness (BHR), allergen positive skin prick test (SPT) and total serum Immunoglobulin E (IgE) from nine Caucasian asthma populations.

**Results:**

Significant evidence for susceptibility loci was identified for quantitative traits including; BHR (989 pedigrees, n = 4,294) 2p12-q22.1, 6p22.3-p21.1 and 11q24.1-qter, allergen SPT (1,093 pedigrees, n = 4,746) 3p22.1-q22.1, 17p12-q24.3 and total IgE (729 pedigrees, n = 3,224) 5q11.2-q14.3 and 6pter-p22.3. Analysis of the asthma phenotype (1,267 pedigrees, n = 5,832) did not identify any region showing genome-wide significance.

**Conclusion:**

This study represents the first linkage meta-analysis to determine the relative contribution of chromosomal regions to the risk of developing asthma and atopy. Several significant results were obtained for quantitative traits but not for asthma confirming the increased phenotype and genetic heterogeneity in asthma. These analyses support the contribution of regions that contain previously identified asthma susceptibility genes and provide the first evidence for susceptibility loci on 5q11.2-q14.3 and 11q24.1-qter.

## Background

Asthma is a disease characterised by recurrent respiratory symptoms, reversible variable airway obstruction, airway inflammation and increased bronchial hyper-responsiveness [1]. Estimates suggest that 100–150 million people worldwide have asthma. Atopy is a predisposition towards the development of immediate hypersensitivity against common environmental antigens. Atopy and asthma are closely related, however they are not interchangeable. Most asthmatic individuals are atopic but atopic individuals may not have asthmatic symptoms. Asthma and atopic disease show strong familial aggregation and heritability estimates vary between 36–79% [2]. A greater understanding of the genetic basis of asthma and atopy holds great promise for the identification of novel therapeutic targets.

Linkage analysis using short tandem repeats or microsatellites to follow the transfer of genetic information between generations has been used to identify chromosomal regions that potentially contain asthma and atopy susceptibility genes. Commonly sub-phenotypes of clinical relevance are used including; elevated total Immunoglobulin E (IgE) levels, atopy defined by positive skin prick test to one or more allergen or elevated specific IgE and bronchial hyper-responsiveness (BHR) [3]. These studies have identified linkage on multiple chromosomal regions *e.g*. 2q22-33, 5q31.1-33, 6p21.3, 11q13, 12q14.3-24.1, 13q14, 14q11.2-13 and 19q13; however replication of linkage findings has been limited [3]. Low statistical power and the potential for type I and type II errors may explain these findings. Combining data has the potential to provide inferences about the consistency of results across studies and to identify regions that contain asthma and atopy susceptibility genes.

The aim of the current study was to complete the first meta-analysis of available genome wide linkage data for asthma and related traits (asthma *per se*, BHR, total IgE, allergen skin prick test response (SPT)) in the Caucasian population using the Genome Scan Meta Analysis (GSMA) method [4]. GSMA is a non parametric, rank based approach and has been used extensively in other disorders *e.g*. schizophrenia [5].

## Methods

### Systematic Literature Search

To identify published studies for inclusion in the GSMA of asthma and related phenotypes we completed a systematic literature review in September 2006. We used PubMed and the following search string (*Asthma OR BHR OR bronchial hyper responsiveness OR bronchial hyperreactivity OR AHR OR airway hyper responsiveness OR respiratory hypersensitivity OR histamine OR slope OR methacholine OR atopy OR atopic OR dermatitis, atopic OR IgE OR immunoglobulin E OR SPT OR skin prick tests OR skin tests) AND linkage AND genome-scan OR scan OR genome OR genomewide OR genome-wide OR LOD OR microsatellite*). Limits were set on the search including; published in English, human studies, published 1996–2006 and the exclusion of reviews. This initial search identified 516 matches of which 488 were discarded as not containing genome-wide linkage data. A further eight studies were discarded as they were in non-Caucasian populations and we wished to avoid any population stratification issues leaving 20 potential Caucasian studies for inclusion. Genome-wide linkage analyses for asthma related traits in the Hutterite Founder population [6] was not included in the current analyses as limited data was available and the focus of the present study was Caucasian out-bred populations.

Of the 20 manuscripts identified a further nine were removed from the analyses for a combination of the following reasons; the study was superseded by another including the families from the original, LOD score plots in the manuscript were not labelled and/or unreadable, no genome-wide data was presented *e.g*. in the manuscript describing the positional cloning of *ADAM33*, linkage analyses in 460 families for asthma, IgE and BHR phenotypes were performed but has never been published in full [7] or the phenotypes studied did not meet our criteria. All authors were contacted and invited to provide complete datasets.

### Phenotype definition and study inclusion/exclusion

There was a large degree of heterogeneity in phenotype definitions and so these were standardised for inclusion. Asthma was defined using doctor diagnosis and/or currently taking asthma medication, however we did include data from the Dutch population which used an algorithm based on asthma symptoms, the presence of BHR, reversibility to β_2_-adrenergic receptor agonist and smoking history to define asthma [8]. Analyses were completed with and without the Dutch families. Total IgE levels were analysed in the genome scans using quantitative data generated by Pharmacia CAP system [9], Pharmacia IgE EIA [10], Phadebas PRIST [11] and ELISA techniques [12,13] which have shown good inter assay correlation [14,15], therefore all studies were included. Positive skin prick response to one or more allergen was used as a marker of atopy and for inclusion in the GSMA. However, allergens used in each study varied; Dermatphagoides pteronyssinus, mixed grass pollen [9], Dermatphagoides pteronyssinus, Cladosporium herbarum, Alternaria tenuis, timothy grass, olive, birch, Parieteria judaica, ragweed, Aspergillus, Blatella Germancia [11], mixed grass and tree pollens, mixed weeds, Dermatphagoides pteronyssinus, dog, cat, a mixture of guinea pig and rabbit, horse, Aspergillus fumigatus, Alternaria alternate [10], house dust mite [12], Dermatphagoides pteronyssinus and 10 others [13] and Dermatphagoides pteronyssinus, D. farinae, dog, cat, grass mix, pollen and alternaria [16]. BHR was measured in multiple ways using different provocation stimuli *e.g*. histamine or methacholine providing categorical and/or quantitative analyses. These provocation stimuli have shown a significant correlation (r = 0.95) in the responses induced [17], however it is worth noting that this has not been reproduced in all studies. Studies with BHR data were included in the GSMA irrespective of the criteria used in the original manuscript.

### Genome Scan Meta Analysis (GSMA)

GSMA was used as it is able to combine linkage data from studies with different marker sets and analysed by different methods including permutated p-values. GSMA was implemented using GSMA software [4,5,18]. Briefly, the genome was divided into 120 bins of approximately 30 cM, for each study the maximum evidence for linkage *e.g*. LOD score or p-value was identified for each bin and these bins were then ranked relative to their evidence for linkage in that study. These ranks were summed across studies and the summed rank (SR) forms the basis of the test statistic [4]. An ordered rank (OR) statistic was also generated which gives a genome wide interpretation of significance by comparing the n-th highest summed rank with the distribution of the n-th highest summed ranks obtained through simulation [5]. We completed an unweighted and weighted analyses using information content (√(no. pedigrees × no. markers)) as a weighting factor.

### Statistical Significance

Simulation studies have shown that any bin with a p(SR) < 0.05 and a p(OR) < 0.05 has a high probability of containing a true susceptibility gene [5]. Applying Bonferroni correction a p < 0.000417 provides evidence for genome wide significance for linkage and a p < 0.0083 provides suggestive evidence for linkage [5].

## Results

### Data included

The selection criteria and data requests provided eleven studies of nine Caucasian asthma populations for our analyses (Table 1) including data from 1,267 pedigrees (n = 5,832) for asthma (80.2% of pedigrees available in the public domain or following request, missing 249 [19] and 65 pedigrees [20]), 989 pedigrees (n = 4,294) for BHR (79.9% of available, missing 249 pedigrees [19]), 1,093 pedigrees (n = 4,746) for SPT (81.5% of available, missing 249 pedigrees [19]) and 729 pedigrees (n = 3,224) for total IgE (65.9% of available, missing 249 [19], and 129 pedigrees [21]).

**Table 1:** Characteristics of genome-wide linkage studies included in the GSMA

		**NUMBER OF.^(b)^**						
						
**COHORT (REF.)**	**PHENOTYPES^(a)^**	**PED. (AFF/IND.)**	**MKS.**	**WF.**	**MKR MAP (SP.)^(c)^**	**STUDY TYPE^(d)^**	**ANALYSIS PROGRAM**	**INCLUDED IN GSMA**
Australian [9]	**B: **Slope (-,0–12 μmol meth.)	80 (25/364)	253	142.267	**- **(10 cM)	Nonpar.two.	-	**B: **23 p-values < 0.05
	**I: **Quantitative							**I: **21 p-values < 0.05
	**S: **2 allergens							**S: **19 p-values < 0.05
CSGA [33]	**A: **Quest./Doc diag	79 (200/316)	360	168.642	Marshfield (10 cM)	Nonpar. multi.	Modified GENEHUNTER	**A: **LOD scores from graphs
German [34]	**B: **PD_20 _(Neb, < 8 mg/ml meth.)	97 (200/415)	333	179.725	Modified Genethon (10.7 cM)	Nonpar. multi.	GENEHUNTER, MAP-MAKER/SIBS	**B: **Complete dataset
French [11]	**A: **Quest./Meds.	46 (102/210)	254	108.093	– (13 cM)	Nonpar. multi.	GENEHUNTER, SIBPAIR	**A: **11 p-values
	**B: **Slope (-, meth.)							**B: **8 p-values
	**I: **Quantitative							**I: **8 p-values
	**S: **11 allergens							**S: **19 p-values
Icelandic [35]	**A: **Doc diag./Meds.	175 (596/1134)	976*	413.280	Decode (4 cM)	Nonpar. multi.	ALLEGRO	**A: **LOD scores from graphs
Dutch [10, 36, 37]	**A: **Algorithm	200 (-/1159)	366	270.555	Marshfield Weber v8 (10 cM)	Nonpar.	SOLAR, GENEHUNTER	**A: **Complete dataset
	**B: **PD_20 _(-, < 32 mg/ml hist.)							**B: **Complete dataset
	**I: **Quantitative							**I: **Complete dataset
	**S: **16 allergens							**S: **Complete dataset
German [12]	**A: **Doc diag.	201 (506/867)	364	270.489	Modified Genethon (10 cM)	Nonpar. multi.	MERLIN	**A: **Complete dataset
	**I: **Quantitative/Catagorical							**I: **Complete quantitative dataset
	**S: **HDM+							**S: **Complete dataset
Australian [13]	**A: **Quest./Meds.	202 (169/591)	624	355.032	- (7.1 cM)	Nonpar. multi.	MERLIN, SOLAR	**A: **Complete dataset
	**B: **PD_20 _(-, < 7.8 μmol hist)							**B: **Complete dataset
	**I: **Quantitative							**I: **Complete dataset
	**S: **11 allergens.							**S: **Complete dataset
GAIN [16]	**A: **Doc diag.	364 (1014/1555)	396*	379.663	Decode (-)	Nonpar. multi.	MERLIN	**A: **LOD scores from graphs
	**B: **PD_20 _(-, < 8 mg/ml meth.)							**B: **LOD scores from graphs
	**S: **7 allergens							**S: **LOD scores from graphs

(a) A = asthma; B = bronchial hyper-responsiveness (Neb = nebuliser, Dos = dosimeter); I = total serum IgE; S = skin prick test response; Quest = questionnaire; Doc diag = doctor diagnosis; Meds = asthma medication; Meth = methacholine; Hist = histamine; HDM = house dust mite; PD_20 _= provocation dose resulting in a 20% fall in FEV_1_. (b) Ped = total genotyped pedigrees; AFF = total genotyped asthmatic cases, Ind = total genotyped individuals; Mks = total autosomal microsatellite markers; Wt = weighting factor. (c) Sp = average marker spacing. (d) Nonpar = nonparametric; Two = two point; Multi = multipoint. (-) = not provided, *number of autosomal markers not given, total number used for weighting.

### Asthma

**Figure 1:**
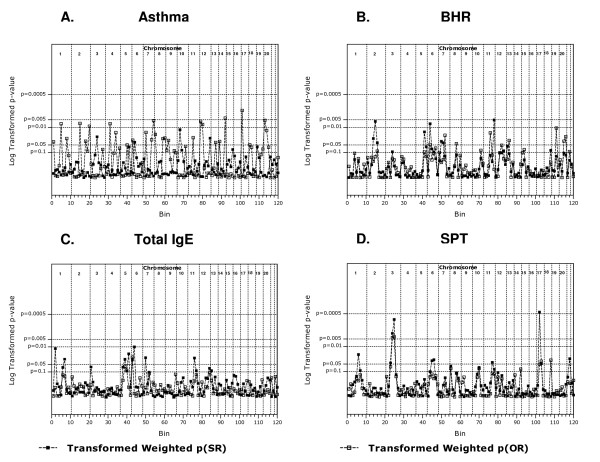
**p(SR) & p(OR) in weighted GSMA. ***A*. asthma. *B*. bronchial hyper-responsiveness. *C*. total serum IgE. *D*. skin prick test response. A p(SR) of p < 0.000417 = significant linkage, p < 0.0083 = suggestive linkage. p(SR) & p(OR) data were transformed using f(x) = 0.05/x and plotted on a log_10 _scale to improve clarity.

**Figure 2:**
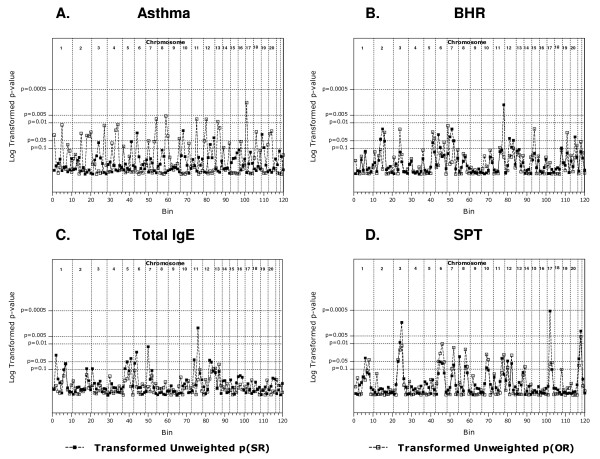
**p(SR) & p(OR) in unweighted GSMA. ***A*. asthma. *B*. bronchial hyper-responsiveness. *C*. total serum IgE. *D*. skin prick test response. A p(SR) of p < 0.000417 = significant linkage, p < 0.0083 = suggestive linkage. p(SR) & p(OR) data were transformed using f(x) = 0.05/x and plotted on a log_10 _scale to improve clarity.

The weighted asthma analyses did not identify any chromosomal region with a p(SR) and p(OR) < 0.05 (Figure 1 and Table 2). No bin p(SR) met genome wide significance (p < 0.000417) or suggestive evidence for linkage (p < 0.0083) in these analyses however three regions demonstrated a p(SR) < 0.05; 6p22.3-p21.1, 10p14-q11.21 and 12q24.31-qter (Table 2). Eight regions met suggestive linkage criteria in the ordered rank analyses; 1p31.1-p13.3, 2p12-q22.1, 4p14-q13.3, 7q34-qter, 12pter-p12,1, 12p12.1-p11.21, 14q32.12-qter, 17pter-p12 and 20pter-p12.3 (Table 2). Analyses of the asthma phenotype using unweighted GSMA generated similar findings to the weighted analyses (Figure 2). To confirm that the inclusion of the Dutch linkage data for the asthma phenotype (defined by algorithm) had not confounded the analyses we completed GSMA without these data focusing on doctor diagnosed asthma only (1,067 pedigrees). Again, no chromosomal region with a p(SR) and p(OR) < 0.05 was identified (data not shown).

**Table 2:** Weighted GSMA for Asthma (1,267 pedigrees, n = 5,832)

					**PROBABILITY**	
					
**BIN^(a)^**	**GENETIC LOCUS^(b)^**	**DISTANCE (cM)^(c)^**	**PHYSICAL POSITION (KB)^(d)^**	**SUMMED RANK (SR)**	**p(SR)**	**p(OR)**
1	1pter-p36.23	0.00–20.61	pter-9332	376.576	0.68121	0.03775
5	1p31.1-p13.3	113.69–142.24	82867–110103	346.834	0.78259	0.00718*
8	1q31.1-q32	201.58–231.11	185232–210150	347.872	0.77937	0.02791
15	2p12-q22.1	101.56–128.41	79617–119705	341.174	0.79974	0.00694*
18	2q31.1-q34	177.53–206.74	172805–212021	317.985	0.86170	0.03548
20	2q35-qter	233.62–269.07	230618-qter	316.169	0.86603	0.00908
24	3p14.1-q12.3	88.60–117.76	64182–103187	612.921	0.02348	0.80059
31	4p14-q13.3	51.60–78.97	38279–72275	343.939	0.79152	0.00714*
34	4q28.3-q32.1	134.74–159.30	137492–160828	346.992	0.78212	0.01609
43	6pter-p22.3	0.00–32.62	pter-16854	383.747	0.65433	0.03220
44	6p22.3-p21.1	32.62–65.14	16854–43207	592.740	0.03905	0.72593
50	7p21.1-p14.1	29.28–59.93	19430–40230	317.779	0.86218	0.01583
53	7q31.1-q34	122.48–148.11	75216–138638	381.650	0.66230	0.03107
54	7q34-qter	148.11–181.97	138638-qter	313.741	0.87152	0.00536*
55	8pter-p22	0.00–27.40	pter-13111	376.368	0.68196	0.01899
59	8q22-q24.21	110.2–137.92	99237–127416	365.804	0.71999	0.02820
60	8q24.21-qter	137.92–167.90	127416-qter	373.141	0.69378	0.02636
62	9p22.3-p21.1	27.32–53.60	14264–29850	349.926	0.77291	0.03321
68	10p14-q11.21	29.15–62.23	10591–36230	635.659	0.01229	0.79026
75	11p12-q13.3	47.06–72.82	36450–70234	353.473	0.76155	0.02578
79	12pter-p12.1	0.00–24.45	pter-11686	299.278	0.90180	0.00570*
80	12p12.1-p11.21	24.45–53.28	11686–32879	292.228	0.91466	0.00766*
84	12q24.31-qter	139.61–170.60	120806-qter	607.388	0.02714	0.65821
87	13q22.2-q33.1	58.54–85.41	74875–102346	354.469	0.75831	0.04007
89	14pter-q13.1	0.00–40.11	pter- 33529	362.366	0.73182	0.03622
92	14q32.12-qter	105.00-138.18	90647-qter	305.539	0.88932	0.00423*
101	17pter-p12	0.00–25.14	pter-11325	312.768	0.87374	0.00213*
105	18pter-p11	0.00–24.08	pter- 7462	377.842	0.67650	0.04965
113	20pter-p12.3	0.00–21.15	pter-7608	339.111	0.80587	0.00524*
114	20p12.3-p11	21.15–47.52	7608–21259	282.935	0.92982	0.01324

Chromosomal regions with a p(SR) or p(OR) < 0.05 are shown. ^a^Bin number from GSMA (1–120 inclusive). ^b^Cytogenetic band (Taken from April 2002 Genome Browser, UCSC). ^c^Genetic distance in Marshfield cM (not cumulative). ^d^Physical position in kilobase pairs (Taken from December 2006 UniSTS, NCBI/Genome Browser, UCSC). *p < 0.000417 = Genome wide significance for linkage, p < 0.0083 = Suggestive for linkage [5].

### Bronchial Hyper-responsiveness

The weighted BHR analyses strongly suggested that 6p22.3-p21.1 contains BHR susceptibility gene(s) as a p(SR) and p(OR) < 0.05 was observed (p = 0.007, p = 0.049 respectively (Figure 1 and Table 3)). Two other regions showed suggestive evidence (p < 0.0083) for linkage to the BHR phenotype; 2p12-q22.1 (p(SR) = 0.006) and 11q24.1-qter (p(SR) = 0.005). In the unweighted analyses three regions showed evidence for linkage (p(SR) and p(OR) ≤ 0.05), *i.e*. 2q22.1-q23.3, 7q12.11-q31.1 and 5q23.2-q34 (Figure 2).

**Table 3:** Weighted GSMA for bronchial hyper-responsiveness (989 pedigrees, n = 4,294)

					**PROBABILITY**	
					
**BIN^(a)^**	**GENETIC LOCUS^(b)^**	**DISTANCE (cM)^(c)^**	**PHYSICAL POSITION (KB)^(d)^**	**SUMMED RANK (SR)**	**p(SR)**	**p(OR)**
14	2p16.2-p12	76.34–101.56	54063–79617	532.855	0.02751	0.22094
15	2p12-q22.1	101.56–128.41	79617–119705	576.585	0.00574*	0.14743
16	2q22.1-q23.3	128.41–154.48	119705–151529	518.593	0.04085	0.08859
41	5q23.2-q34	131.48–164.19	123774–162087	551.701	0.01511	0.09586
**44**	**6p22.3-p21.1**	**32.62–65.14**	**16854–43207**	**571.381**	0.00716*	**0.04861**
50	7p21.1-p14.1	29.28–59.93	19430–40230	523.367	0.03597	0.24658
51	7p14.1-q21.11	59.93–91.67	40230–81002	518.746	0.04070	0.19114
52	7q21.11-q31.1	91.67–122.48	81002–109766	461.380	0.13926	0.02065
58	8q13.1-q22	82.84–110.20	67744–99237	468.188	0.12312	0.04443
76	11q13.3-q22.3	72.82–98.98	70185–103732	465.589	0.12913	0.01652
78	11q24.1-qter	123.00–147.77	122977-qter	579.520	0.00505*	0.46485
86	13q13.2-q22.2	26.87–58.54	33636–74875	472.999	0.11255	0.03312
111	19q12-q13.33	52.59–75.41	35843–54589	460.759	0.14078	0.01070
115	20p11-q13.13	47.52–72.27	21259–46747	476.308	0.10563	0.03503
116	20q13.13-qter	72.27–101.22	46747-qter	467.593	0.12448	0.02343

Chromosomal regions with a p(SR) or p(OR) < 0.05 are shown. ^a^Bin number from GSMA (1–120 inclusive). ^b^Cytogenetic band (Taken from April 2002 Genome Browser, UCSC). ^c^Genetic distance in Marshfield cM (not cumulative). ^d^Physical position in kilobase pairs (Taken from December 2006 UniSTS, NCBI/Genome Browser, UCSC). *p < 0.000417 = Genome wide significance for linkage, p < 0.0083 = Suggestive for linkage [5].

### Positive allergen skin prick test

Weighted analyses of the SPT phenotype identified two regions that had a p(SR) and p(OR) ≤ 0.05 strongly suggesting these regions are susceptibility loci (Figure 1 and Table 4). These regions are 17p12-q24.3 (two adjacent bins, 17p12-q21.33 p(SR) = 0.00043, p(OR) = 0.050 and 17q21.33-q24.3 p(SR) = 0.047, p(OR) = 0.038) and region 3p22.1-q22.1 (three adjacent bins, 3p22.1-p14.1 p(SR) = 0.045, p(OR) = 0.063, 3p14.1-q12.3 p(SR) = 0.003, p(OR) = 0.0045 and 3q12.3-q22.1 p(SR) = 0.00084, p(OR) = 0.00406). The analyses of the unweighted SPT datasets identified chromosomes 3 and 17 as containing the major determinants (Figure 2).

**Table 4:** Weighted GSMA for skin prick test response (1,093 pedigrees, n = 4,746)

					**PROBABILITY**	
					
**BIN^(a)^**	**GENETIC LOCUS^(b)^**	**DIST. (cM)^(c)^**	**PHYS POS. (KB)^(d)^**	**SUMMED RANK (SR)**	**p(SR)**	**p(OR)**
6	1p13.3-q23.3	142.24–170.84	110103–159125	541.271	0.02081	0.22906
**23**	**3p22.1-p14.1**	**63.12–88.60**	**38845–64182**	**513.869**	**0.04488**	**0.06344**
**24**	**3p14.1-q12.3**	**88.60–117.76**	**64182–103187**	**591.392**	0.00298*	**0.00450**
**25**	**3q12.3-q22.1**	**117.76–146.60**	**103187–134474**	**614.373**	0.00084*	**0.00406**
45	6p21.1-q15	65.14–99.01	43207–90985	521.717	0.03654	0.12598
46	6q15-q23.2	99.01–131.07	90985–132584	525.276	0.03318	0.18839
77	11q22.3-q24.1	98.98–123.00	103732–122977	515.746	0.04276	0.11460
**102**	**17p12-q21.33**	**25.14–63.62**	**11325–39726**	**624.777**	0.00043*	**0.05044**
**103**	**17q21.33-q24.3**	**63.62–93.98**	**39726–66600**	**511.187**	**0.04799**	**0.03824**
108	18q22.1-qter	96.48–126.00	60025-qter	209.839	0.96132	0.03480
118	21q21.3-qter	25.26–57.77	27903-qter	528.536	0.03029	0.28738

Chromosomal regions with a p(SR) or p(OR) < 0.05 are shown. ^a^Bin number from GSMA (1–120 inclusive). ^b^Cytogenetic band (Taken from April 2002 Genome Browser, UCSC). ^c^Genetic distance in Marshfield cM (not cumulative). ^d^Physical position in kilobase pairs (Taken from December 2006 UniSTS, NCBI/Genome Browser, UCSC). *p < 0.000417 = Genome wide significance for linkage, p < 0.0083 = Suggestive for linkage [5].

### Total Immunoglobulin E

Weighted analyses of the IgE phenotype strongly suggested 5q11.2-q14.3 (p(SR) = 0.031, p(OR) = 0.060) and 6pter-p22.3 (p(SR) = 0.033, p(OR) = 0.026) contain genes that influence IgE levels (Figure 1 and Table 5). The region adjacent to 6pter-p22.3, *i.e*. 6p22.3-p21.1 has a p(SR) = 0.00999 approaching suggestive linkage providing further evidence for this region. Analyses of the IgE phenotype using the unweighted GSMA showed similar overall findings (Figure 2).

**Table 5:** Weighted GSMA for total serum IgE (729 pedigrees, n = 3,224)

					**PROBABILITY**	
	
**BIN^(a)^**	**GENETIC LOCUS^(b)^**	**DIST. (cM)^(c)^**	**PHYS POS. (KB)^(d)^**	**SUMMED RANK (SR)**	**p(SR)**	**p(OR)**
2	1p36.23-p35.3	20.61–54.30	9332 – 24723	476.251	0.01171	0.41867
7	1q23.3-q31.1	170.84–201.58	159125–185232	450.659	0.03099	0.14664
**39**	**5q11.2-q14.3**	**64.14–97.82**	**55758–88765**	**450.303**	**0.03127**	**0.06031**
41	5q23.2-q34	131.48–164.19	123774–162087	464.611	0.01890	0.40276
**43**	**6pter-p22.3**	**0.00–32.62**	**pter-16854**	**448.903**	**0.03276**	**0.02583**
44	6p22.3-p21.1	32.62–65.14	16854–43207	479.723	0.00999	0.71984
50	7p21.1-p14.1	29.28–59.93	19430–40230	454.706	0.02708	0.41494
76	11q13.3-q22.3	72.82–98.98	70185–103732	453.763	0.02792	0.22926

Chromosomal regions with a p(SR) or p(OR) < 0.05 are shown. ^a^Bin number from GSMA (1–120 inclusive). ^b^Cytogenetic band (Taken from April 2002 Genome Browser, UCSC). ^c^Genetic distance in Marshfield cM (not cumulative). ^d^Physical position in kilobase pairs (Taken from December 2006 UniSTS, NCBI/Genome Browser, UCSC). *p < 0.000417 = Genome wide significance for linkage, p < 0.0083 = Suggestive for linkage [5].

### Multiple phenotype analyses

In addition to the primary phenotype analyses we investigated overlapping chromosomal regions containing genetic determinants of asthma and asthma related traits consistent with gene(s) having pleiotrophic effects in asthma and allergy (Table 6). Interestingly, region 6p22.3-p21.1 which contains the HLA region showed a p(SR) < 0.05 in the asthma, BHR and IgE analyses potentially as expected due to the role of HLA restriction in many immunological mechanisms. Several other regions also showed overlapping concordance, in particular regions; 3p14.1-q12.3 (asthma, SPT), 5q23.2-q34 (asthma, BHR, IgE) and 7p21.1-14.1 (asthma, BHR, IgE).

**Table 6:** Overlapping chromosomal regions in weighted GSMA for asthma, and the three intermediate phenotypes

				**ASTHMA**		**BHR**		**TOTAL IGE**		**SPT RESPONSE**	
							
**BIN^(a)^**	**GENETIC LOCUS^(b)^**	**DIST. (cM)^(c)^**	**PHYS POS. (KBp)^(d)^**	**p(SR)**	**p(OR)**	**p(SR)**	**p(OR)**	**p(SR)**	**p(OR)**	**p(SR)**	**p(OR)**
6	1p13.3-q23.3	142.24–170.84	110103–159125	-	-	-	-	0.06630	0.13155	0.02081	0.22906
7	1q23.3-q31.1	170.84–201.58	159125–185232	-	-	-	-	0.03099	0.14664	0.08924	0.13402
15	2p12-q22.1	101.56–128.41	79617–119705	0.79974	0.00694	0.00574*	0.14743	-	-	-	-
24	3p14.1-q12.3	88.60–117.76	64182–103187	0.02348	0.80059	0.09312	0.17837	-	-	0.00298	0.00450*
40	5q5q14.3-q23.2	97.82–131.48	88765–123774	0.71879	0.05004	-	-	0.09195	0.08596	-	-
41	5q23.2-q34	131.48–164.19	123774–162087	0.06027	0.77886	0.01511	0.09586	0.01890	0.40276	-	-
43	6pter-p22.3	0.00–32.62	pter-16854	0.65433	0.03220	-	-	0.03276	0.02583	-	-
44	6p22.3-p21.1	32.62–65.14	16854–43207	0.03905	0.72593	0.00716*	0.04861	0.00999	0.71984	0.09934	0.16043
45	6p21.1-q15	65.14–99.01	43207–90985	-	-	0.07128	0.19832	-	-	0.03654	0.12598
46	6q15-q23.2	99.01–131.07	90985–132584	-	-	0.09445	0.10925	-	-	0.03318	0.18839
50	7p21.1-p14.1	29.28–59.93	19430–40230	0.86218	0.01583	0.03597	0.24658	0.02708	0.41494	-	-
55	8pter-p22	0.00–27.40	pter-13111	0.68196	0.01899	-	-	-	-	0.06027	0.07341
71	10q23.33-q26.13	117.42–142.78	95985–123274	0.85805	0.05664	0.16985	0.08106	-	-	-	-
76	11q13.3-q22.3	72.82–98.98	70185–103732	-	-	0.12913	0.01652	0.02792	0.22926	-	-
77	11q22.3-q24.1	98.98–123.00	103732–122977	-	-	-	-	0.08890	0.12887	0.04276	0.11460
78	11q24.1-qter	123.00–147.77	122977-qter	-	-	0.00505*	0.46485	-	-	0.08072	0.12542
84	12q24.31-qter	139.61–170.60	120806-qter	0.02714	0.65821	-	-	0.07175	0.10909	-	-
85	13pter-q13.2	0.00–26.87	pter-33636	-	-	0.05521	0.18686	0.08835	0.22042	-	-
92	14q32.12-qter	105.00–138.18	90647-qter	0.88932	0.00423*	-	-	-	-	0.95708	0.07887
108	18q22.1-qter	96.48–126.00	60025-qter	-	-	0.08190	0.24216	-	-	0.96132	0.03480
115	20p11-q13.13	47.52–72.27	21259–46747	0.65424	0.06184	0.10563	0.03503	-	-	-	-
118	21q21.3-qter	25.26–57.77	27903-qter	-	-	0.20402	0.09140	-	-	0.03029	0.28738

Chromosomal regions with p(SR) or p(OR) < 0.1 in two or more weighted GSMA are shown. ^a^Bin number from GSMA (1–120 inclusive). ^b^Cytogenetic band (Taken from April 2002 Genome Browser, UCSC). ^c^Genetic distance in Marshfield cM (not cumulative). ^d^Physical position in kilobase pairs (Taken from December 2006 UniSTS, NCBI/Genome Browser, UCSC). *p < 0.000417 = Genome wide significance for linkage, p < 0.0083 = Suggestive for linkage.

## Discussion

This study represents the first meta-analysis of asthma and related trait linkage data using the majority of the data available for the Caucasian asthma cohorts in the public domain. This analysis combines data from 10 years of asthma and atopy genetics and is extremely timely providing a definitive analysis of available linkage data to complement the highly anticipated whole genome association findings. Analysis of asthma and atopy quantitative traits identified significant evidence for relatively few chromosomal regions as containing susceptibility gene(s) using the most stringent genome-wide criteria *i.e*. BHR (6p22.3-p21.1), total IgE (5q11.2-q14.3 and 6pter-p22.3) and positive allergen skin prick test (3p22.1-q22.1, 17p12-q24.3). Significantly no chromosomal region met stringent genome-wide criteria in the asthma phenotype analyses. This study did provide supporting evidence for regions containing previously identified asthma susceptibility genes.

Linkage analyses has proven to be highly successful in single gene disorders *e.g*. cystic fibrosis but has been problematic in asthma and atopy mainly due to the complex genetic basis of these phenotypes and the use of inadequate samples sizes leading to both type I and type II errors. In the current study we aimed to combine all available linkage data for asthma and related trait phenotypes (BHR, total IgE, positive allergen skin prick test) and provide inferences about the consistency of results across studies, ultimately providing a focus for future gene discovery. The analyses of the quantitative traits provided the most significant findings and may be consistent with the observation that using objective markers of disease adds to the homogeneity of the data and may improve results. In addition the number of genes regulating these phenotypes may be smaller than "asthma" and power to find these may be increased.

This study strongly suggested that regions 17p12-q21.33 and 3p14.1q22.1 contain gene(s) that influence allergen skin prick responses and by inference atopy. Both of these regions are large spanning 28.5 and 70.3 Mbp respectively. The 3p21 region has been identified as containing genetic determinants of specific allergen responses in the Hutterite founder asthma cohort [22] and has been identified as an atopic dermatitis locus (3p24-22) [23]. Linkage to chromosome 17 and specific allergen responses has been described in the Hutterite population however these linkages map to 17q25 in asthma [22]. Linkage to atopic dermatitis on 17q23.1 has been reported [23]. Also of significance is the fact that the chromosome 17 locus (17p12-q21.33) identified in the current analyses contains the recently identified *ORMDL3 *gene [24]. Using whole genome association variants in the *ORMDL3 *gene were shown to be associated with childhood onset asthma [24]. Overall our data suggest that the major genes influencing allergen skin prick responses are found on chromosomes 3 and 17.

In the total IgE analyses there was strong evidence for the presence of genes(s) regulating IgE production in the 5q11.2-q14.3 and 6pter-p21.1 regions. This region on chromosome 6 contains the Human Leukocyte Antigen (HLA) locus and so may be predicted to contain determinants of immunological processes. The finding that 5q11.2-q14.3 may contain gene(s) that influence IgE production is novel and warrants further investigation. The IgE analyses also confirmed the potential contribution of genes within the 5q23.2-q34 and 11q13.3-q22.3 regions that have previously been suggested [25]. Interestingly, of the four positionally cloned genes identified using IgE as a key phenotype only the region encompassing the *GPRA *gene showed limited (non significant) linkage (7p21.1-p14.1, p(SR) = 0.027).

In the BHR analyses the 6p22.3-p21.1 region was identified as containing susceptibility gene(s). This region contains the HLA locus and the HLA-G gene within this region has previously been identified as a potential asthma and BHR susceptibility gene using four cohorts (including the Dutch cohort used in the current analyses) [26]. Our data confirms this region as a BHR locus and less significantly a potential asthma locus (p(SR) = 0.039). Interestingly, four of the six populations used for the BHR analyses ranked the chromosome 2p locus identified in the top 33% of bins including the Genetics of Asthma International Network (GAIN) study (data not shown). Further mapping of the 2p locus in the GAIN population using single nucleotide polymorphisms (SNP) spanning the region refined the linkage peak to ~70 cM with the greatest evidence being for the BHR phenotype (LOD score 4.58) [16]. Region 2p12-q22.1 contains the *DPP10 *gene that has previously been identified as an asthma and total IgE susceptibility gene [27] and the *IL1RN *gene identified using asthma as the primary phenotype [28]. The identification of 11q24.1-qter as a potential BHR susceptibility locus appears to be novel and therefore these data may provide a platform for novel BHR susceptibility gene identification. The BHR analyses also confirmed the potentially modest contribution of other loci in determining the BHR phenotype including *e.g*. 5q23.2-q34 and 19q12-q13.33. In the analyses of the BHR phenotype significant linkage was not driven by studies using a specific agonist *i.e*. studies using both methacholine and histamine provocation contributed to the signal at a specific locus (data not shown). These data suggest responsiveness to these agents share a common genetic basis and provide support for combining studies in the meta-analyses using these different provocation stimuli.

Significantly, using asthma as a phenotype we did not identify any chromosomal region as showing genome-wide significance in our analyses. In most studies, asthma was defined as a doctor's diagnosis. In the Dutch study, families were ascertained through a proband with a doctor's diagnosis of asthma. In the offspring of these asthma patients, an algorithm was used since a doctor's diagnosis *per se *underestimated the prevalence of asthma in the offspring [8]. To confirm that the inclusion of the Dutch linkage data for the asthma phenotype had not confounded the analyses we completed GSMA without these data and again no chromosomal region with a p(SR) and p(OR) < 0.05 was identified (data not shown). These data may reflect the true locus heterogeneity in asthma or reflect differences in phenotype definition when comparing asthma based on doctors' diagnosis over different cohorts. In addition the use if a binary trait *i.e*. affection will have the lowest intrinsic power compared to continual data *e.g*. IgE levels.

Interestingly, the recently published whole genome association study using 994 asthmatic children and 1,243 non asthmatic children identified only 16 SNPs (eight on chromosome 17) from a total of 317,447 SNPs tested showing a significant association with asthma *per se *(5% false discovery threshold, stratification corrected) [24]. This study complements our analysis using data from 1,257 families and taken together suggests that the use of asthma as a phenotype may be confounded due to locus heterogeneity in asthma and/or issues concerning phenotype definition/heterogeneity when combining cohorts. It is important to note that the family based studies included in this meta-analyses address the genetic basis of asthma defined in children *i.e*. the mean age of siblings in most studies is < 16 years.

Several regions showed suggestive evidence for linkage to the asthma phenotype mainly based on the p(OR) statistic, however caution should be taken interpreting p(OR) in isolation, especially in the presence of incomplete data sets [5]. Further evidence for the chromosome 12 and 20 loci comes from the finding that adjacent bins have a p(OR) < 0.05 suggesting the linkage is spanning the bin interval. 4/6 regions containing the positionally cloned asthma susceptibility genes *i.e. ADAM33 *(20p13[7]), *PHF11 *(13q14.3 [29]), *DPP10 *(2q14.1 [27]), *HLAG *(6p21.3 [26]), *GPRA *(7p14.3 [30]), *IL1RN *(2q13 [28]) and the recently reported gene *ORMDL3 *(17q12-q21[24]) were identified by the GSMA approach, *ADAM33 *(p(OR) = 0.005), *DPP10 *and *IL1RN *(p(OR) = 0.007) and less significantly *HLA-G *(p(SR) = 0.039) and *GRPA *(p(OR) = 0.031). In addition our data also suggests that further investigation of additional chromosomal regions may be productive *e.g*. 1p31.1-p13.3 and 14q32.12-qter. Recently, the 1p31 and 14q32 regions have been highlighted as potential asthma loci in a French cohort with data suggesting 1p31 may contain gene(s) of importance to asthma and atopic dermatitis co morbidity and the 14q32 locus may interact with smoking exposure and contain asthma susceptibility gene(s) [31,32].

The analysis of overlap between chromosomal regions confirmed the importance of the HLA locus on chromosome 6 as being a key susceptibility locus in asthma and also highlighted other regions that may be of importance *i.e*. 5q23.2-q34 and 7p21.1-14.1. The 5q23.2-q34 region contains the cytokine gene cluster (*IL4, Il13, IL5, IL12B*) and has previously been suggested as an asthma/atopy susceptibility locus [3] and the 7p21.1-14.1 region contains the previously identified asthma susceptibility gene, *GPRA *(7p14.3) [30].

In conclusion, we present the first systematic meta-analyses of asthma and related trait linkage data in the Caucasian population. These data are based on the majority of the data available in the public domain (or through collaboration) therefore we do not consider that exclusion or missing data for other populations has biased our analyses. While the GSMA method has limitations *e.g*. only large chromosomal regions can be identified, these analyses have determined the role of several previously identified susceptibility loci and highlighted the significance of regions not previously implicated in asthma and atopy susceptibility. Importantly, this study also highlighted the limitations of using asthma as a phenotype in contrast to quantitative traits even with the increased power of 1,267 families composed of 5,832 individuals. Finally, these data will provide useful guidance for the interpretation of the anticipated genome wide association analyses in asthma and atopy.

## List of abbreviations

GSMA: Genome Scan Meta-analysis; SPT: allergen positive skin prick test; IgE: total serum Immunoglobulin E (IgE); BHR: bronchial hyper-responsiveness (BHR).

## Competing interests

The authors declare that they have no competing interests.

## Authors' contributions

IS designed the study, compiled and interpreted results and wrote the manuscript. SD contributed to the study design, completed the data analyses and contributed to the writing of the manuscript. GHK, MW and MAF provided datasets, contributed to the design of the study and the writing of the manuscript. JB and IPH contributed to the design of the study and the writing of the manuscript. All authors read and approved the final manuscript.

## References

[B1] Tattersfield AE, Knox AJ, Britton JR, Hall IP (2002). Asthma. Lancet.

[B2] Los H, Koppelman GH, Postma DS (1999). The importance of genetic influences in asthma. Eur Respir J.

[B3] Ober C, Hoffjan S (2006). Asthma genetics 2006: the long and winding road to gene discovery. Genes Immun.

[B4] Wise LH, Lanchbury JS, Lewis CM (1999). Meta-analysis of genome searches. Ann Hum Genet.

[B5] Levinson DF, Levinson MD, Segurado R, Lewis CM (2003). Genome scan meta-analysis of schizophrenia and bipolar disorder, part I: Methods and power analysis. Am J Hum Genet.

[B6] Ober C, Tsalenko A, Parry R, Cox NJ (2000). A second-generation genomewide screen for asthma-susceptibility alleles in a founder population. Am J Hum Genet.

[B7] Van Eerdewegh P, Little RD, Dupuis J, Del Mastro RG, Falls K, Simon J, Torrey D, Pandit S, McKenny J, Braunschweiger K, Walsh A, Liu Z, Hayward B, Folz C, Manning SP, Bawa A, Saracino L, Thackston M, Benchekroun Y, Capparell N, Wang M, Adair R, Feng Y, Dubois J, FitzGerald MG, Huang H, Gibson R, Allen KM, Pedan A, Danzig MR, Umland SP, Egan RW, Cuss FM, Rorke S, Clough JB, Holloway JW, Holgate ST, Keith TP (2002). Association of the ADAM33 gene with asthma and bronchial hyperresponsiveness. Nature.

[B8] Panhuysen CI, Bleecker ER, Koeter GH, Meyers DA, Postma DS (1998). Characterization of obstructive airway disease in family members of probands with asthma. An algorithm for the diagnosis of asthma. Am J Respir Crit Care Med.

[B9] Daniels SE, Bhattacharrya S, James A, Leaves NI, Young A, Hill MR, Faux JA, Ryan GF, le Souef PN, Lathrop GM, Musk AW, Cookson WO (1996). A genome-wide search for quantitative trait loci underlying asthma. Nature.

[B10] Koppelman GH, Stine OC, Xu J, Howard TD, Zheng SL, Kauffman HF, Bleecker ER, Meyers DA, Postma DS (2002). Genome-wide search for atopy susceptibility genes in Dutch families with asthma. J Allergy Clin Immunol.

[B11] Dizier MH, Besse-Schmittler C, Guilloud-Bataille M, Annesi-Maesano I, Boussaha M, Bousquet J, Charpin D, Degioanni A, Gormand F, Grimfeld A, Hochez J, Hyne G, Lockhart A, Luillier-Lacombe M, Matran R, Meunier F, Neukirch F, Pacheco Y, Parent V, Paty E, Pin I, Pison C, Scheinmann P, Thobie N, Vervloet D, Kauffmann F, Feingold J, Lathrop M, Demenais F (2000). Genome screen for asthma and related phenotypes in the French EGEA study. Am J Respir Crit Care Med.

[B12] Altmuller J, Seidel C, Lee YA, Loesgen S, Bulle D, Friedrichs F, Jellouschek H, Kelber J, Keller A, Schuster A, Silbermann M, Wahlen W, Wolff P, Schlenvoigt G, Ruschendorf F, Nurnberg P, Wjst M (2005). Phenotypic and genetic heterogeneity in a genome-wide linkage study of asthma families. BMC Pulm Med.

[B13] Ferreira MA, O'Gorman L, Le Souef P, Burton PR, Toelle BG, Robertson CF, Visscher PM, Martin NG, Duffy DL (2005). Robust estimation of experimentwise P values applied to a genome scan of multiple asthma traits identifies a new region of significant linkage on chromosome 20q13. Am J Hum Genet.

[B14] Bayne NK, Mathews KP (1982). Determination of total IgE by ELISA in tubes and plates compared with PRIST. Clinical biochemistry.

[B15] Bousquet J, Chanez P, Chanal I, Michel FB (1990). Comparison between RAST and Pharmacia CAP system: a new automated specific IgE assay. J Allergy Clin Immunol.

[B16] Pillai SG, Chiano MN, White NJ, Speer M, Barnes KC, Carlsen K, Gerritsen J, Helms P, Lenney W, Silverman M, Sly P, Sundy J, Tsanakas J, von Berg A, Whyte M, Varsani S, Skelding P, Hauser M, Vance J, Pericak-Vance M, Burns DK, Middleton LT, Brewster SR, Anderson WH, Riley JH (2006). A genome-wide search for linkage to asthma phenotypes in the genetics of asthma international network families: evidence for a major susceptibility locus on chromosome 2p. Eur J Hum Genet.

[B17] Sekerel BE, Saraclar Y, Kalayci O, Cetinkaya F, Tuncer A, Adalioglu G (1997). Comparison of four different measures of bronchial responsiveness in asthmatic children. Allergy.

[B18] Pardi F, Levinson DF, Lewis CM (2005). GSMA: software implementation of the genome search meta-analysis method. Bioinformatics.

[B19] Bouzigon E, Dizier MH, Krahenbuhl C, Lemainque A, Annesi-Maesano I, Betard C, Bousquet J, Charpin D, Gormand F, Guilloud-Bataille M, Just J, Le Moual N, Maccario J, Matran R, Neukirch F, Oryszczyn MP, Paty E, Pin I, Rosenberg-Bourgin M, Vervloet D, Kauffmann F, Lathrop M, Demenais F (2004). Clustering patterns of LOD scores for asthma-related phenotypes revealed by a genome-wide screen in 295 French EGEA families. Hum Mol Genet.

[B20] Colilla S, Nicolae D, Pluzhnikov A, Blumenthal MN, Beaty TH, Bleecker ER, Lange EM, Rich SS, Meyers DA, Ober C, Cox NJ (2003). Evidence for gene-environment interactions in a linkage study of asthma and smoking exposure. J Allergy Clin Immunol.

[B21] Mathias RA, Freidhoff LR, Blumenthal MN, Meyers DA, Lester L, King R, Xu JF, Solway J, Barnes KC, Pierce J, Stine OC, Togias A, Oetting W, Marshik PL, Hetmanski JB, Huang SK, Ehrlich E, Dunston GM, Malveaux F, Banks-Schlegel S, Cox NJ, Bleecker E, Ober C, Beaty TH, Rich SS (2001). Genome-wide linkage analyses of total serum IgE using variance components analysis in asthmatic families. Genet Epidemiol.

[B22] Ober C, Tsalenko A, Willadsen S, Newman D, Daniel R, Wu X, Andal J, Hoki D, Schneider D, True K, Schou C, Parry R, Cox N (1999). Genome-wide screen for atopy susceptibility alleles in the Hutterites. Clin Exp Allergy.

[B23] Bradley M, Soderhall C, Luthman H, Wahlgren CF, Kockum I, Nordenskjold M (2002). Susceptibility loci for atopic dermatitis on chromosomes 3, 13, 15, 17 and 18 in a Swedish population. Hum Mol Genet.

[B24] Moffatt MF, Kabesch M, Liang L, Dixon AL, Strachan D, Heath S, Depner M, von Berg A, Bufe A, Rietschel E, Heinzmann A, Simma B, Frischer T, Willis-Owen SA, Wong KC, Illig T, Vogelberg C, Weiland SK, von Mutius E, Abecasis GR, Farrall M, Gut IG, Lathrop GM, Cookson WO (2007). Genetic variants regulating ORMDL3 expression contribute to the risk of childhood asthma. Nature.

[B25] Hoffjan S, Ober C (2002). Present status on the genetic studies of asthma. Curr Opin Immunol.

[B26] Nicolae D, Cox NJ, Lester LA, Schneider D, Tan Z, Billstrand C, Kuldanek S, Donfack J, Kogut P, Patel NM, Goodenbour J, Howard T, Wolf R, Koppelman GH, White SR, Parry R, Postma DS, Meyers D, Bleecker ER, Hunt JS, Solway J, Ober C (2005). Fine mapping and positional candidate studies identify HLA-G as an asthma susceptibility gene on chromosome 6p21. Am J Hum Genet.

[B27] Allen M, Heinzmann A, Noguchi E, Abecasis G, Broxholme J, Ponting CP, Bhattacharyya S, Tinsley J, Zhang Y, Holt R, Jones EY, Lench N, Carey A, Jones H, Dickens NJ, Dimon C, Nicholls R, Baker C, Xue L, Townsend E, Kabesch M, Weiland SK, Carr D, von Mutius E, Adcock IM, Barnes PJ, Lathrop GM, Edwards M, Moffatt MF, Cookson WO (2003). Positional cloning of a novel gene influencing asthma from chromosome 2q14. Nat Genet.

[B28] Gohlke H, Illig T, Bahnweg M, Klopp N, Andre E, Altmuller J, Herbon N, Werner M, Knapp M, Pescollderungg L, Boner A, Malerba G, Pignatti PF, Wjst M (2004). Association of the interleukin-1 receptor antagonist gene with asthma. Am J Respir Crit Care Med.

[B29] Zhang Y, Leaves NI, Anderson GG, Ponting CP, Broxholme J, Holt R, Edser P, Bhattacharyya S, Dunham A, Adcock IM, Pulleyn L, Barnes PJ, Harper JI, Abecasis G, Cardon L, White M, Burton J, Matthews L, Mott R, Ross M, Cox R, Moffatt MF, Cookson WO (2003). Positional cloning of a quantitative trait locus on chromosome 13q14 that influences immunoglobulin E levels and asthma. Nat Genet.

[B30] Laitinen T, Polvi A, Rydman P, Vendelin J, Pulkkinen V, Salmikangas P, Makela S, Rehn M, Pirskanen A, Rautanen A, Zucchelli M, Gullsten H, Leino M, Alenius H, Petays T, Haahtela T, Laitinen A, Laprise C, Hudson TJ, Laitinen LA, Kere J (2004). Characterization of a common susceptibility locus for asthma-related traits. Science.

[B31] Dizier MH, Bouzigon E, Guilloud-Bataille M, Genin E, Oryszczyn MP, Annesi-Maesano I, Demenais F (2007). Evidence for a locus in 1p31 region specifically linked to the co-morbidity of asthma and allergic rhinitis in the EGEA study. Hum Hered.

[B32] Dizier MH, Bouzigon E, Guilloud-Bataille M, Siroux V, Lemainque A, Boland A, Lathrop M, Demenais F (2007). Evidence for gene x smoking exposure interactions in a genome-wide linkage screen of asthma and bronchial hyper-responsiveness in EGEA families. Eur J Hum Genet.

[B33] CSGA (1997). A genome-wide search for asthma susceptibility loci in ethnically diverse populations. The Collaborative Study on the Genetics of Asthma (CSGA). Nat Genet.

[B34] Wjst M, Fischer G, Immervoll T, Jung M, Saar K, Rueschendorf F, Reis A, Ulbrecht M, Gomolka M, Weiss EH, Jaeger L, Nickel R, Richter K, Kjellman NI, Griese M, von Berg A, Gappa M, Riedel F, Boehle M, van Koningsbruggen S, Schoberth P, Szczepanski R, Dorsch W, Silbermann M, Wichmann HE (1999). A genome-wide search for linkage to asthma. German Asthma Genetics Group. Genomics.

[B35] Hakonarson H, Bjornsdottir US, Halapi E, Palsson S, Adalsteinsdottir E, Gislason D, Finnbogason G, Gislason T, Kristjansson K, Arnason T, Birkisson I, Frigge ML, Kong A, Gulcher JR, Stefansson K (2002). A major susceptibility gene for asthma maps to chromosome 14q24. Am J Hum Genet.

[B36] Meyers DA, Postma DS, Stine OC, Koppelman GH, Ampleford EJ, Jongepier H, Howard TD, Bleecker ER (2005). Genome screen for asthma and bronchial hyperresponsiveness: interactions with passive smoke exposure. J Allergy Clin Immunol.

[B37] Xu J, Postma DS, Howard TD, Koppelman GH, Zheng SL, Stine OC, Bleecker ER, Meyers DA (2000). Major genes regulating total serum immunoglobulin E levels in families with asthma. Am J Hum Genet.

